# FRAILTY AND DEVELOPMENT ACROSS INDIAN STATES

**DOI:** 10.1093/geroni/igad104.3051

**Published:** 2023-12-21

**Authors:** Benjamin Seligman, Arunika Agarwal, David Bloom

**Affiliations:** UCLA School of Medicine, Los Angeles, California, United States; Harvard TH Chan School of Public Health, Boston, Massachusetts, United States; Harvard TH Chan School of Public Health, Boston, Massachusetts, United States

## Abstract

Introduction Frailty, a common syndrome of vulnerability among older adults, provides insight into the overall health of aging populations. Life-course influences play an important role in late-life health, but the role of social and economic development has not been well-explored. We study these indicators at the state level in the Longitudinal Aging Study in India (LASI).

Methods: Participants in LASI were adults aged 45 years or older from 2017-19 in 35 of 36 states and union territories. Frailty was assessed by a 48-item deficit accumulation frailty index (FI). Robust was defined as FI of 0 to 0.1, pre-frail was >0.1 – 0.2, mildly frail >0.2 – 0.3, moderately frail >0.3 – 0.4, and severely frail was >0.4. Development indicators were GDP per capita, infant mortality rate (IMR), and literacy rate in 1981 and 2018 (2011 for literacy), from the Reserve Bank of India. Associations were assessed using beta regression. Results IMR in 1981 had a small but significant association with frailty (OR 1.00 [1.00-1.00]). For GDP per capita and literacy, 1981 values were associated with greater frailty (1.15 [1.10-1.21] and 1.01 [1.01-1.01] respectively). However, 2018/2011 values were associated with lower frailty (0.94 [0.93-0.95] and 0.99 [0.99-1.00]). These associations were similar after stratifying by age group. Discussion Associations between frailty and state-level development varied both by indicator and by time of assessment. Sensitivity to the environment these indicators measure – such as IMR – may differ by age. Whether that sensitivity leads to protection or vulnerability may also change with age.

